# Summary of the mpox outbreak in Canada, April 28−December 31, 2022

**DOI:** 10.14745/ccdr.v51i23a04

**Published:** 2025-02-12

**Authors:** Meera Bhulabhai, Jeyasakthi Venugopal, Mireille Plamondon, Geneviève Bergeron, Geneviève Cadieux, Jesse Kancir, Mayank Singal, Katherine Twohig, Austin Zygmunt, Erin Schillberg, Rukshanda Ahmad, Julia Paul

**Affiliations:** 1 Emergency Management Branch, Public Health Agency of Canada, Toronto, ON; 2 Infectious Diseases and Vaccination Programs Branch, Public Health Agency of Canada, Ottawa, ON; 3 Direction régionale de santé publique de Montréal, Montréal, QC; 4 Nova Scotia Health Authority, Halifax, NS; 5 BC Centre for Disease Control, Vancouver, BC; 6 Public Health Ontario, Toronto, ON; 7 Data, Surveillance and Foresight Branch, Public Health Agency of Canada, Ottawa, ON

**Keywords:** mpox, communicable diseases, zoonoses, orthopoxvirus, disease outbreaks, men who have sex with men

## Abstract

**Background:**

Mpox is an infectious disease caused by the monkeypox virus (MPXV), closely related to the virus that causes smallpox. In May 2022, cases of mpox were reported in previously non-endemic countries including Canada.

**Objective:**

To summarize the epidemiology of the mpox outbreak in Canada, as well as key public health response activities, between April and December 2022.

**Methods:**

The Public Health Agency of Canada (PHAC) worked closely with local, provincial and territorial public health authorities to develop national case investigation and reporting tools, including national case definitions for confirmed and probable mpox cases. Based on de-identified case data submitted to PHAC, patterns and trends were examined, including the distribution of cases by sociodemographic, clinical and transmission factors.

**Results:**

Overall, 1,474 mpox cases (1,396 confirmed, 78 probable) were reported to PHAC. All reported cases were associated with MPXV clade IIb. Mpox disproportionately affected gay, bisexual and other men who have sex with men (80.0%) and those between 20−49 years of age (86.0%). Available data suggests that the most likely mode of disease acquisition was through sexual contact, with limited evidence on other possible modes of transmission. Some cases were hospitalized (3.0%); however, there were no mpox-related deaths in Canada.

**Conclusion:**

Rapid coordination and surveillance activities supported the timely implementation of tailored interventions, including the procurement and distribution of vaccines. These actions, coupled with vaccination uptake and behavioural changes, contributed to reducing transmission and health impacts of mpox on the Canadian population.

## Introduction

Mpox (formerly monkeypox) is a viral infectious disease caused by the monkeypox virus (MPXV), which is a species within the *Orthopoxvirus* genus. First discovered in non-human primates in 1958, human cases of this disease experience symptoms akin to those of smallpox but with a much lower case fatality rate ((1)). From the 1970s to the early 2000s, the epidemiological range of MPXV remained mostly limited to central and western Africa, given the proximity to its wildlife reservoir, including rodents and other small mammals ((1)). The occasional detection of cases outside the traditional endemic range of MPXV was mainly due to travel and exportation of reservoir animals ((2–6)). However, increased transmission among humans has been observed in the last two decades, likely relating to changes at the human-environment interface causing increased zoonotic spillovers; reduction in cross-protection from the smallpox vaccine following the end of global vaccination programs; and genetic evolution of MPXV ((7)).

On May 16, 2022, the United Kingdom reported a cluster of mpox cases among gay, bisexual and other men who have sex with men (GBMSM), without a history of travel to an endemic area ((8)). In Canada, the city of Montréal in Québec investigated the first cases of mpox between May 8−13, 2022 ((9)), and this cluster of cases was then confirmed by the Public Health Agency of Canada’s (PHAC) National Microbiology Laboratory on May 19, 2022 ((10)). The Public Health Agency of Canada conducted a preliminary risk assessment, escalated its Health Portfolio Operations Centre to a Level 2 (Increased Vigilance and Readiness) on May 21, 2022, and activated an Incident Management System to respond to the emergence of mpox and support the coordination of the outbreak investigation and response activities across the country, in collaboration with local, provincial and territorial (LPT) public health authorities. We conducted a descriptive analysis of mpox cases reported in Canada between April and December 2022, and examined differences in sociodemographic, clinical and transmission characteristics between GBMSM and non-GBMSM subgroups.

## Methods

### Case definitions, data collection and investigation

Following the initial reporting of mpox cases in Canada, PHAC collaborated closely with affected local and provincial public health units and interim guidance issued by the World Health Organization ((11)) to develop national case definitions (see **Table 1**) ((12)) and investigation tools ((13)). This work relied on epidemiological and clinical observations made by public health units in the affected regions, as well as data shared by international partners. This rapid knowledge exchange from affected regions informed the development and harmonization of standardized tools for public health investigations across the country. Local, provincial and territorial public health authorities conducted case investigations and data were shared with PHAC on a weekly basis. Through case investigations, LPT public health authorities collected information on demographics, symptoms and clinical manifestations, as well as information on relevant risk factors, including recent sexual and travel history. Local, provincial and territorial public health authorities conducted their own molecular testing to screen or confirm cases whenever possible and were supported by National Microbiology Laboratory for confirmatory testing and genomic sequencing, as needed.

**Table 1:** National mpox case classifications in Canada, 2022

Case classification	Definition
Confirmed	A person who is laboratory-confirmed for monkeypox virus by detection of unique sequences of viral DNA either by real-time polymerase chain reaction and/or sequencing
Probable	A person of any age who presents with an unexplained^a^ acute rash or lesion(s)^b^ AND Has one or more of the following: · An epidemiological link^c^ to a probable or confirmed mpox case in the 21 days before symptom onset, OR · Reported travel history to or place of residence in a location where mpox is reported^d^ in the 21 days before symptom onset
Suspected	A person of any age who presents with one or more of the following: · An unexplained^a^ acute rash^b^ AND has at least one of the following signs or symptoms: o Headache o Acute onset of fever (higher than 38.5°C) o Lymphadenopathy (swollen lymph nodes) o Myalgia (muscle and body aches) o Back pain o Asthenia (profound weakness) · An unexplained^a^ acute genital, perianal or oral lesion(s)

^a^ Other common causes of acute rash can include varicella zoster, herpes zoster, measles, herpes simplex, syphilis, chancroid, lymphogranuloma venereum and hand-foot-and-mouth disease

^b^ Acute rash: mpox illness includes a progressively developing rash that usually starts on the face and then spreads elsewhere on the body. The rash can affect the mucous membranes in the mouth, tongue and genitalia. The rash can also affect the palms of hands and soles of the feet. The rash can last for two to four weeks and progresses through the following stages: macules, papules, vesicles, pustules and scabs. Note: It is not necessary to obtain negative laboratory results for listed infectious causes of rash in order to classify a case as suspected

^c^ An epidemiological link can be: face-to-face exposure, including health workers without appropriate personal protective equipment (PPE); direct physical contact, including sexual contact; or contact with contaminated materials, such as clothing or bedding

^d^ Reported travel history includes regional, national or international travel in the 21 days before symptom onset to any area where mpox may be reported

## Interventions

The Public Health Agency of Canada coordinated various responses to the 2022 mpox outbreak in Canada. A critical component of PHAC’s response was the rapid deployment of medical countermeasures from existing stockpiles, primarily Imvamune® (Modified Vaccinia Ankara Bavarian Nordic), a vaccine originally approved for immunization against smallpox. The expansion of the vaccine’s indication to include immunization against all orthopoxviruses for adults considered at high risk for exposure had been granted by Health Canada in 2020, which allowed the rapid implementation of a vaccination program in response to the mpox outbreak. Local, provincial and territorial public health authorities initially offered one dose of the two-dose vaccine schedule to eligible individuals to strategically leverage the limited vaccine supply and maximize vaccination uptake. Following early recommendations issued by the Québec Immunization Committee, Montréal Public Health in Québec initiated the first post-exposure prophylaxis vaccination program for mpox on May 30, 2022, which was later expanded to include pre-exposure prophylaxis on June 14, 2022 ((9)). The National Advisory Committee on Immunization recommended the use of Imvamune for prophylaxis in the context of mpox outbreaks in Canada on June 8, 2022 ((14)), which supported the rapid implementation of vaccination programs against mpox in all other jurisdictions. The Public Health Agency of Canada continued to procure additional Imvamune vaccine doses to support program expansion of vaccination activities in all jurisdictions; worked closely with LPTs to establish harmonized, national guidance for health professionals ((13)) as well as case and contact management ((15)); issued travel health advisories; and disseminated tailored risk communication through official, press and social media platforms. The Public Health Agency of Canada’s activation was de-escalated to normal operations on December 15, 2022, following a sustained reduction in mpox case reports across Canada.

## Data analyses

People with a confirmed or probable mpox infection with illness onset between April 28, 2022, and December 31, 2022, were included for the analysis. An epidemic curve was developed to visually summarize aggregate case numbers over time by epidemiological date, which was defined as the earliest available date from the following hierarchy: date of symptom onset, date of specimen collection for laboratory testing and date reported to the local public health unit. Descriptive statistics were computed to summarize case patterns by sociodemographic, clinical and transmission characteristics. Additionally, differences were examined across GBMSM and non-GBMSM subgroups. Statistically significant differences in case patterns between GBMSM and non-GBMSM were determined based on a t-test for continuous variables and a chi-square or Fisher’s exact test for categorical variables. Given the low number of probable cases, confirmed and probable mpox cases were examined together for all analyses. Information on sexual behaviours was not directly collected through case report forms but was derived based on available data from the following variables: sex, gender and gender(s) of sexual partner(s) in the 21 days before the date of symptom onset. Epidemiological link refers to a known case’s contact with another possible or known case or contact with contaminated material. Data are presented as counts and percentages (%). All analyses were conducted in R statistical software ((16,17)).

## Results

**Figure 1:**
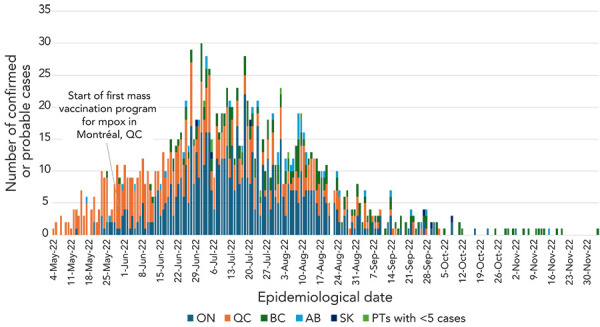
Distribution of confirmed or probable mpox cases in Canada by earliest date and province/territory, April–December 2022 Abbreviations: AB, Alberta; BC, British Columbia; ON, Ontario; PTs, provinces/territories in Canada; QC, Québec; SK, Saskatchewan

A total of 1,474 mpox cases were reported to PHAC in 2022 (1,396 confirmed; 78 probable). The first two cases of mpox were confirmed on May 19, 2022, with the first reported case having a symptom onset date of April 28, 2022. Based on available information from laboratory testing and genomic sequencing, all mpox cases reported in 2022 were associated with MPXV clade IIb. As shown in **Figure 1**, there was a steady increase in cases, which included two peaks in the summer of 2022. The majority of cases were reported in Ontario, Québec and British Columbia, accounting for 96% of all cases (**Table 2**). No cases were reported in Prince Edward Island, Nunavut and the Northwest Territories. There was a notable decrease in cases starting in August 2022, and by October 2022, cases were more sporadic.

**Table 2:** Summary of mpox cases reported in Canada, 2022

Characteristic	n (%)	Unknown, n (%)
Total cases	1,474 (100%)	N/A
Confirmed	1,396 (94.7%)	
Probable	78 (5.3%)	
**Sex**		
Male	757 (98.7%)	707 (48.0%)
Female	9 (1.2%)	
**Gender**		
Woman	11 (0.8%)	152 (10.3%)
Man	1,296 (98.0%)	
Transgender	4 (0.3%)	
Non-binary	3 (0.2%)	
Other	7 (0.5%)	
**Sexual orientation**		
GBMSM	1,184 (80.3%)	244 (16.6%)
Non-GBMSM	46 (3.1%)	
**Province**		
Ontario	700 (47.5%)	0 (0.0%)
Québec	525 (35.6%)	
British Columbia	193 (13.1%)	
Alberta	43 (2.9%)	
Saskatchewan	6 (0.4%)	
Newfoundland and Labrador	2 (0.1%)	
Yukon	2 (0.1%)	
Manitoba	1 (0.1%)	
New Brunswick	1 (0.1%)	
Nova Scotia	1 (0.1%)	

Abbreviations: GBMSM, gay, bisexual and other men who have sex with men; non-GBMSM, all other sexual identities and behaviours, including those for whom we have no information on sex/gender and gender(s) of sexual partner(s); N/A, not applicable

Available data on sex and gender was as follows: 99.0% (n=757/767) of cases were of male sex (Table 2) and 98.0% (n=1,296/1,322) identified as men. Based on available information on sex, gender and gender(s) of sexual partner(s), 80.3% (n=1,184/1,474) were grouped as GBMSM, 3.1% (n=46/1,474) as non-GBMSM and information regarding the gender(s) of sexual partner(s) was unknown for 16.6% (n=244/1,474). The majority of cases were between 20−49 years old (86.1%, n=1,269/1,474), with a mean age of 37 years. There were no significant differences in the distribution of cases by age group between the GBMSM and non-GBMSM groups (**Table 3**). Nearly one-third of cases with available data (30.8%, n=299/972) reported living with HIV; a significantly higher proportion of people reported living with HIV in the GBMSM group (31.0%, n=246/793) compared to the non-GBMSM group (13.2%, n=5/38). Additionally, 7.0% (n=48/244) of cases for whom we have no information on the gender(s) of sexual partner(s) reported living with HIV. A diagnosis of a concurrent sexually transmitted or blood-borne infection was reported among 22.2% (n=209/941) of cases with information available, with 22.7% (n=173/763) in the GBMSM group and 20.6% (n=7/34) in the non-GBMSM group.

**Table 3:** Frequency and percent distribution of people with a confirmed or probable diagnosis of mpox by select demographic and clinical factors across subgroups^a^

Case characteristics	Overall (N=1,474)	GBMSM (n=1,184)	Non-GBMSM (n=46)	*p*-value
	n (%)			
**Age group (in years)**				
<15	2 (0.1%)	N/A	N/A	0.281^b^
15–19	8 (0.5%)	6 (0.5%)	0 (0.0%)	
20–29	345 (23.4%)	271 (22.9%)	19 (41.3%)	
30–39	579 (39.3%)	475 (40.1%)	12 (26.1%)	
40–49	345 (23.4%)	276 (23.3%)	9 (19.6%)	
50–59	136 (9.2%)	111 (9.4%)	3 (6.5%)	
60–69	53 (3.6%)	41 (3.5%)	3 (6.5%)	
≥70	5 (0.3%)	0 (0.0%)	0 (0.0%)	
**Hospitalized**				
Yes	46 (3.4%)	35 (3.1%)	2 (4.3%)	0.651^c^
No	1,319 (96.6%)	1,106 (96.9%)	44 (95.7%)	
**Admitted to an ICU**				
Yes	3 (1.1%)	3 (1.2%)	0 (0.0%)	1.000^c^
No	277 (98.9%)	244 (98.8%)	14 (100%)	
**HIV status**				
Positive	299 (30.8%)	246 (31.0%)	5 (13.2%)	0.019^a^
Negative	673 (69.2%)	547 (69.0%)	33 (86.8%)	
**Concurrent STBBI**				
Yes	209 (22.2%)	173 (22.7%)	7 (20.6%)	0.776
No	731 (77.8%)	590 (77.3%)	27 (79.4%)	
**Received vaccination**				
Yes	307 (45.2%)	276 (47.4%)	2 (11.8%)	0.004^a^
No	372 (54.8%)	306 (52.6%)	15 (88.2%)	
**Most common symptoms reported**				
Rash/lesion	1,245 (89.1%)	1,061 (91.5%)	41 (89.1%)	0.588^c^
Fever	744 (76.2)	634 (75.5%)	24 (85.7%)	0.213
Chills	394 (75.2%)	331 (74.7%)	14 (82.4%)	0.580^c^
Lymphadenopathy	706 (73.2%)	610 (73.3%)	21 (65.6%)	0.336
Fatigue/exhaustion	699 (73.0%)	597 (72.0%)	23 (79.3%)	0.388
**Site of lesion**				
Anogenital	483 (75.0%)	424 (76.4%)	14 (70.0%)	0.592^c^
Face	197 (30.6%)	172 (31.0)	7 (35.0%)	0.704
Tongue/mouth/lip	89 (13.8%)	78 (14.1%)	4 (20.0%)	0.510^c^
Limbs	237 (36.8%)	199 (35.9%)	12 (60.0%)	0.028^a^
Hand	153 (23.8%)	126 (22.7%)	6 (30.0%)	0.425^c^
Feet	67 (10.4%)	52 (9.4%)	5 (25.0%)	0.039^a,b^
Torso	186 (28.9%)	161 (29.0%)	8 (40.0%)	0.289
**Number of lesions**				
≤1	33 (15.1%)	28 (15.0%)	1 (16.7%)	0.868^b^
2–9	126 (57.5%)	110 (58.8%)	3 (50.0%)	
10–49	53 (24.2%)	45 (24.1%)	2 (33.3%)	
50–99	5 (2.28%)	3 (1.6%)	0 (0.0%)	
≥100	2 (0.9%)	1 (0.5%)	0 (0.0%)	
**Common exposures and likely mode of acquisition**				
Contact with a possible or known case/contaminated material	191 (59.1%)	162 (57.9%)	3 (50.0%)	0.700^b^
Person-to-person transmission via sexual contact	717 (96.2%)	642 (97.1%)	16 (80.0%)	0.003^a,b^
Travel history in the 21 days prior to symptom onset	276 (22.1%)	241 (22.1%)	8 (17.8%)	0.489

Abbreviations: GBMSM, gay, bisexual and other men who have sex with men; ICU, intensive care unit; non-GBMSM, all other sexual identities and behaviours, including those for whom we have no information on sex/gender and gender(s) of sexual partner(s); N/A, not applicable; STBBI, sexually transmitted and blood-borne infection

^a^ Statistically significant (*p*<0.05) difference between GBMSM and non-GBMSM mpox cases. The *p*-value was determined based on a χ^2^ test of homogeneity at α=0.05

^b^ The *p*-value determined based on an independent t-test to compare mean age (in years) between GBMSM and non-GBMSM mpox cases

^c^ The *p*-value determined based on Fisher’s exact test instead of a χ^2^ test given that expected cell values are less than five

Among the GBMSM group, sexual contact was the most likely mode of acquisition for 97.1% (n=642/661) of cases, and 57.9% (n=162/280) of cases were epidemiologically linked. Among the non-GBMSM group with available data, sexual contact was the most likely mode of acquisition for 80.0% (n=16/20) of cases, whilst 50.0% (n=3/6) had a known epidemiological link.

The majority of cases provided information on recent travel history during the 21 days before symptom onset (84.8%, n=1,250/1,474), with 22.1% (n=276/1,250) reporting travel outside their province of residence. Among those with travel outside their province of residence, 37.7% (n=104/276) reported international travel, 36.6% (n=101/276) reported domestic travel and 5.1% (n=14/276) reported both international and domestic travel.

Of all cases with information available, 45.2% (n=307/679) reported receiving a vaccination for one or more of the following: previous smallpox vaccination unrelated to the current outbreak; pre-exposure prophylaxis for the current outbreak; or post-exposure prophylaxis for the current outbreak. The proportion of people who received a vaccination was significantly higher among GBMSM (47.4%, n=276/582) compared to the non-GBMSM group (11.8%, n=2/17), reflecting vaccination eligibility criteria, which were based on the epidemiology within the context of the outbreak in Canada.

The five most common symptoms reported among cases with available data were rash/lesions (89.1%, n=1,245/1,398), fever (76.2%, n=744/977), chills (75.2%, n=394/524), lymphadenopathy (73.2%, n=706/964) and fatigue/exhaustion (73.0%, n=699/958). There were no significant differences in common symptom presentation between the GBMSM and non-GBMSM groups. The most common site for a rash was the anogenital/perianal area (75.0%, n=483/1,474). A significantly higher proportion of cases in the non-GBMSM group reported a rash on their limbs (60.0%, n=12/20) and feet (25.0%, n=5/20) compared to the GBMSM group (35.9% and 9.4%, respectively). Among those with information on the number of lesions, the majority of cases reported two to nine lesions (57.5%, n=126/219).

Of all cases for whom information was available, 3.4% (n=46/1,365) were hospitalized, and 1.0% (n=3/300) were admitted to an intensive care unit. Among the GBMSM group, 3.1% of cases (n=35/1,141) were hospitalized, and 4.3% (n=2/46) of non-GBMSM cases were hospitalized; no statistically significant difference in hospitalization between GBMSM and non-GBMSM groups was observed.

## Discussion

This paper describes the epidemiology of the multi-jurisdictional mpox outbreak in Canada between April and December 2022. The 2022 mpox outbreak in Canada predominantly affected GBMSM in large metropolitan cities (Toronto, Montréal and Vancouver) and sexual contact was the likely route of transmission for most cases ((9,18,19)). Similar to other affected countries during the 2022 global mpox outbreak ((20)), initial transmission within high-contact sexual networks likely drove the rapid increase in cases in June 2022. Across both the GBMSM and non-GBMSM groups, the most common symptoms were rash/lesions, fever, chills, lymphadenopathy and fatigue/exhaustion. A higher proportion of cases among GBMSM were living with HIV, compared to non-GBMSM, which is similar to findings from the 2022 global mpox outbreak ((20)). However, there was no statistically significant difference in hospitalization status between the two groups, and the majority of overall cases did not require hospitalization, suggesting low disease severity within the Canadian context. No mpox-related deaths were reported in Canada throughout the outbreak.

Our data also show that the non-GBMSM group had a relatively lower percentage of cases associated with sexual transmission than the GBMSM group. Additionally, presentation of a rash on limbs and feet were significantly more common among non-GBMSM compared to GBMSM, which may suggest variable sites of inoculation and lesion patterns by subgroup. A global case series examining transmission in women and non-binary individuals across 15 countries during the 2022 mpox outbreak reported that acquisition through close household and occupational contacts were more common among this group, compared to sexual contact ((21)). In Canada, there were no reported cases of occupational transmission and a limited number of cases among household contacts, including children younger than 15 years. Given the limited number of non-GBMSM cases in Canada, it is difficult to draw conclusions about other possible modes of transmission.

The outbreak was contained using multi-level approaches with efforts across various levels of government, relying heavily on existing liaisons with local public health units that were most affected by the outbreak, as well as community engagement and advocacy. At the federal level, PHAC: 1) activated an Incident Management System aimed at coordinating and responding to the emerging mpox outbreak, 2) conducted risk assessments to evaluate the domestic situation, 3) recommended actions grounded in evidence and 4) coordinated national meetings to share data and information, collaborate on products and disseminate best practices to respond effectively to the outbreak. At the local and provincial/territorial level, successful strategies included mass pre-exposure prophylaxis vaccination clinics, case and contact management, guidance to health care providers and use of antivirals for clinically severe cases ((9)). Local, provincial and territorial public health authorities engaged with community organizations (e.g., Gay Men’s Sexual Health Alliance in Ontario) to help mobilize affected communities with mpox-related knowledge (e.g., signs and symptoms, mpox vaccination, testing resources, safer sex messaging, etc.) and encourage testing in the presence of compatible symptoms through social media and popular dating applications.

While targeted vaccination played an important role in curbing the mpox outbreak ((22,23)), evidence from mathematical models calibrated to both Canadian case and vaccination data from 2022 highlighted the importance of changes to sexual practices as a major driver in decreasing transmission and the duration of the outbreak ((24)).

Sporadic detection of mpox cases continue to occur globally with small, localized increases in activity occurring in many jurisdictions, including Canada, since 2022 ((25,26)). It is, therefore, important that jurisdictions continue their surveillance efforts for mpox to ensure early detection of a resurgence in cases, including new outbreaks linked to different MPXV lineages, and to continue to offer vaccination against mpox for those who are eligible to minimize transmission and severity of disease ((22,23)). Mpox is a notifiable disease in most provinces and territories across Canada, and in August 2024, it became a nationally notifiable disease, which facilitates ongoing surveillance work. Public Health Agency of Canada, along with federal/LPT partners, are continuing to collaborate and conduct surveillance activities, including laboratory-based and wastewater surveillance of MPXV ((27)).

## Limitations

Due to the complexity of rapidly establishing enhanced surveillance to characterize an emerging infection, there are several considerations regarding the quality and consistency of reported data. First, the development of the national case report form was iterative, with initial cases potentially missing some information that was later prioritized (e.g., expanded data capture for gender). Second, due to the diversity of public health systems across LPTs, some information could not be collected or was incomplete. While a history of vaccination against mpox may have mitigated the extent and severity of the overall clinical presentation of cases ((23)), a high level of missing data for vaccination history (54%) in the national mpox dataset precluded an assessment of this variable in this report. Some data, such as risk factors and likely source of acquisition, were also self-reported, which could have been impacted by recall bias as well as stigma. Lastly, it is likely that the number of mpox cases reported is an underestimate of the true burden of disease as those with mild symptoms who did not seek health care would not have been tested for MPXV.

## Conclusion

The patterns and characteristics of the mpox outbreak in Canada were similar to other countries implicated in the 2022 global mpox outbreak, whereby GBMSM were disproportionately impacted. Changes in sexual practices and uptake of vaccinations helped to rapidly reduce transmission of mpox during the 2022 outbreak in Canada. Alongside partners, PHAC will continue to vigilantly monitor for cases and use evidence-informed practices to support the timely implementation of public health interventions to reduce transmission.
